# Structurally Modified Curcumin Analogs Inhibit STAT3 Phosphorylation and Promote Apoptosis of Human Renal Cell Carcinoma and Melanoma Cell Lines

**DOI:** 10.1371/journal.pone.0040724

**Published:** 2012-08-10

**Authors:** Matthew A. Bill, Courtney Nicholas, Thomas A. Mace, Jonathan P. Etter, Chenglong Li, Eric B. Schwartz, James R. Fuchs, Gregory S. Young, Li Lin, Jiayuh Lin, Lei He, Mitch Phelps, Pui-Kai Li, Gregory B. Lesinski

**Affiliations:** 1 Department of Internal Medicine, The Ohio State University, Columbus, Ohio, United States of America; 2 Division of Medicinal Chemistry and Pharmacognosy, College of Pharmacy, The Ohio State University, Columbus, Ohio, United States of America; 3 Center for Biostatistics, Arthur G. James Cancer Hospital and Richard J. Solove Research Institute, The Ohio State University, Columbus, Ohio, United States of America; 4 Center for Childhood Cancer, The Research Institute at Nationwide Children's Hospital, Department of Pediatrics, College of Medicine, Columbus, Ohio, United States of America; Bauer Research Foundation, United States of America

## Abstract

The Janus kinase-2 (Jak2)-signal transducer and activator of transcription-3 (STAT3) pathway is critical for promoting an oncogenic and metastatic phenotype in several types of cancer including renal cell carcinoma (RCC) and melanoma. This study describes two small molecule inhibitors of the Jak2-STAT3 pathway, FLLL32 and its more soluble analog, FLLL62. These compounds are structurally distinct curcumin analogs that bind selectively to the SH2 domain of STAT3 to inhibit its phosphorylation and dimerization. We hypothesized that FLLL32 and FLLL62 would induce apoptosis in RCC and melanoma cells and display specificity for the Jak2-STAT3 pathway. FLLL32 and FLLL62 could inhibit STAT3 dimerization *in vitro*. These compounds reduced basal STAT3 phosphorylation (pSTAT3), and induced apoptosis in four separate human RCC cell lines and in human melanoma cell lines as determined by Annexin V/PI staining. Apoptosis was also confirmed by immunoblot analysis of caspase-3 processing and PARP cleavage. Pre-treatment of RCC and melanoma cell lines with FLLL32/62 did not inhibit IFN-γ-induced pSTAT1. In contrast to FLLL32, curcumin and FLLL62 reduced downstream STAT1-mediated gene expression of IRF1 as determined by Real Time PCR. FLLL32 and FLLL62 significantly reduced secretion of VEGF from RCC cell lines in a dose-dependent manner as determined by ELISA. Finally, each of these compounds inhibited *in vitro* generation of myeloid-derived suppressor cells. These data support further investigation of FLLL32 and FLLL62 as lead compounds for STAT3 inhibition in RCC and melanoma.

## Introduction

Renal cell carcinoma (RCC) represents an increasing cause of cancer morbidity and mortality worldwide. In the United States alone, 58,240 new cases and over 13,040 deaths were reported in the year 2010 [1]. Approximately 33% of patients present with metastatic disease while of those initially treated by surgical resection, about 40–50% will develop recurrent metastatic disease. RCC is now thought to encompass several histologic subtypes of disease. The most common, and representing about 70% of patients is the clear cell subtype [2].

A greater understanding of the molecular biology of advanced RCC has led to an increased ability to manage this disease with novel therapeutic agents. In addition to immunotherapy with exogenous cytokines [3], pro-angiogenic vascular endothelial growth factor (VEGF) and mammalian target of rapamycin (mTOR) pathways have been deemed relevant therapeutic targets for this disease [4]–[9]. Therefore, recent trials have demonstrated that agents designed to target these pathways, including sorafenib, sunitinib, bevacizumab, everolimus, temsirolimus and pazopanib prolong progression free survival. These studies have led to the approval of these agents for treatment of metastatic RCC by the FDA and EMA [10], [11]. These data highlight the potential for targeted therapy in the management of RCC, and set the precedent for novel combination therapies, as drug resistance remains a legitimate concern for this disease.

The Jak2-STAT3 pathway is emerging as a target of interest for many cancers including RCC and many other forms of cancer including malignant melanoma, the most deadly form of skin cancer [12]. In malignant cells, STAT3 can promote cell proliferation, angiogenesis and inhibit apoptosis. Importantly, constitutive STAT3 activation has been documented in human RCC tumors and has a negative impact on prognosis [13], [14]. A number of experimental strategies targeting the Jak2/STAT3 pathway have been shown to enhance the anti-tumor effects of immune-based therapies in pre-clinical tumor models [15]–[19]. These data suggest that inhibitors of the Jak2-STAT3 pathway deserve further investigation as a novel targeted therapeutic approach for cancer therapy.

Our group has previously described FLLL32, a small molecule inhibitor that can interact with the SH2 domain of the STAT3 protein to selectively inhibit its phosphorylation and dimerization. This lead compound was modeled based on the molecular structure of the natural product, curcumin when locked into its diketone form [20], [21]. The FLLL32 lead compound is unique in comparison to other Jak2-STAT3 pathway inhibitors by virtue of its ability to target both Jak2 and STAT3, and its distinct specificity for STAT3, but not other homologous STAT proteins [20].

In the present study, we describe how the design of this lead compound has undergone further refinement to incorporate more hydrophilic groups, as it retained some structural properties of curcumin, which limits its solubility and bioavailability. Importantly, structural changes in the compound improved its *in vitro* solubility profile, but did not compromise anti-tumor potency or STAT3 specificity. Our data demonstrate that both the FLLL32 lead compound and its structurally modified analog, FLLL62 are specific inhibitors of the Jak2-STAT3 pathway, which induce apoptosis and down-regulate soluble VEGF production in human RCC cell lines. Finally, the FLLL32 and FLLL62 compounds could inhibit *in vitro* generation of myeloid-derived suppressor cells (MDSC). These data suggest that structural modification of the FLLL32 scaffold can be performed in a manner that retains much of the potency and specificity of the lead compound. These compounds can therefore serve as a valid molecular platform on which to optimize and develop improved STAT3-specific inhibitors for cancer therapy.

## Materials and Methods

### Cell Culture and Reagents

Human ACHN RCC, Caki RCC, A375 melanoma, and Hs294T melanoma cell lines were purchased from the American Type Culture Collection (ATCC, Manassas, VA) and cultured according to specifications. Human SK-RC-45 and SK-RC-54 human RCC cell lines were generously provided by Dr. Charles Tannenbaum (Cleveland Clinic Foundation, Cleveland, OH). All cell lines were confirmed free of mycoplasma using the MycoScope mycoplasma PCR detection kit per manufacturer's instructions (Genlantis, San Diego, CA). FLLL32 and FLLL62 were synthesized in Dr. Pui-Kai Li's laboratory as described [20], [21]. Peripheral blood mononuclear cells (PBMCs) were isolated from source leukocytes of healthy adult donors (American Red Cross, Columbus, OH) via density gradient centrifugation with Ficoll-Paque (Amersham Pharmacia Biotech, Uppsala, Sweden) as previously described [22]–[24]. For negative selection of T cells, an additional step using Rosette Sep reagents per manufacturer's instructions was employed (Stem Cell Technologies, Vancouver, BC). Curcumin was purchased from Sigma Aldrich (St. Louis, MO). Recombinant human IFN-γ was purchased from R & D Systems, Inc. (Minneapolis, MN). Recombinant human GM-CSF, IL-6 and IL-2 were purchased from Peprotech (Rocky Hill, NJ). For fluorescence polarization assays, STAT3 protein (>90% purity) was purchased from Abcam (Cambridge, MA). The fluorescent peptide with the amino acid sequence 5-carboxyfluorescein-SpYLPQTV (5-FAM-SpYLPQTV) (97.7% purity) was prepared by Genscript (Piscataway, NJ). The unlabeled peptide with the amino acid sequence SpYLPQTV (95% purity) was prepared by Genscript (Piscataway, NJ). These peptides correspond to the phosphorylated C-terminal region of the STAT3 protein. The fluorescent peptide buffer conditions were 10 mM HEPES, 50 mM NaCl, 1 mM EDTA, 0.1% Nonidet P-40, 2 mM DTT, pH 7.6.

### Docking of FLLL32 and FLLL62

AutoDock version 4.0.0 [25] was used for the docking simulation. We selected the Lamarckian genetic algorithm (LGA) for ligand conformational searching because it has enhanced performance relative to simulated annealing or the simple genetic algorithm. For both compounds, all hydrogens were added and Gasteiger charges were assigned, then non-polar hydrogens were merged. 80×100×70 3-D affinity grids centered on the empty binding site with 0.375 Å spacing were calculated for each of the following atom types: a) protein: A (aromatic C), C, HD, N, NA, OA, SA; b) ligand: C, A, OA, HD, e (electrostatic) and d (desolvation) using Autogrid4. The ligand's translation, rotation and internal torsions are defined as its state variables and each gene represents a state variable. LGA adds local minimization to the genetic algorithm, enabling modification of the gene population. The docking parameters were as follows: trials of 100 dockings, population size of 250, random starting position and conformation, translation step ranges of 2.0 Å, rotation step ranges of 50°, elitism of 1, mutation rate of 0.02, crossover rate of 0.8, local search rate of 0.06, and 50 million energy evaluations. Final docked conformations were clustered using a tolerance of 1.5 Å root-mean-square deviations (RMSD).

### Fluorescence polarization assay development and optimization

The STAT3 fluorescence polarization assay and its corresponding buffer conditions were developed similarly to what has been previously reported [26], [27] using fluorescent and unlabeled peptides identical to those used in prior published studies [26]. The assay was performed in black 384-well microplates (Perkin Elmer, Waltham, MA) in a total volume of 25 µL per well. Measurements were taken with a FlexStation 3 microplate reader (Molecular Devices, Sunnyvale, CA). The fluorescence and fluorescence polarization values were recorded using an excitation filter at 480 nm and an emission filter at 530 nm. The concentration of fluorescent peptide to be used for binding experiments was determined by taking fluorescence and fluorescence polarization readings of various concentrations of 5-FAM-SpYLPQTV in fluorescent peptide buffer [28]. At a concentration of 4 nM 5-FAM-SpYLPQTV the fluorescence readings indicated a 10-fold increase in intensity (tracer/background) and the fluorescence polarization readings indicated a 573.5 mP window (data not shown). This concentration of fluorescent peptide (4 nM) was used for the binding experiments and is similar to what has been used in other studies [26], [27], [29]. To verify that the assay was functioning properly, two standard saturation curves were run; one with increasing concentrations of STAT3 protein (0 to 1000 nM) in the presence of only 4 nM 5-FAM-SpYLPQTV and another where 8 nM of the unlabeled peptide was also present. This method is similar to what has been previously reported [26]. In other studies the concentration of STAT3 was held constant while the concentration of unlabeled peptide was varied [27], [29]. Readings were taken in at least triplicates after a 30–90 minute incubation at room temperature [27], [29]. The IC_50_ values of test compounds were not able to be determined due to solubility issues and high internal fluorescence of test compounds at higher concentrations (data not shown). Instead, similar to what was reported by Hao and colleagues [26], the competitive efficiencies of FLLL32 and FLLL62 were determined by simultaneously running three saturation curves (up to 1020 nM STAT3): one where no inhibitor was present, one in the presence of 50 µM FLLL32, and one in the presence of 50 µM FLLL62. Compounds were diluted in fluorescent peptide buffer from 20 mM DMSO stock solutions (the final amount of DMSO per well was 0.25%). Six measurements were taken after a 24 hour incubation at room temperature in order to allow for complete equilibration. Schust and Berg have previously demonstrated the stability of binding between labeled peptide and STAT3 over a 24 hour period [27]. The K_d_ values were determined and compared to analyze the competitive efficiencies of FLLL32 and FLLL62. The specific binding in these experiments was defined as the contribution to signal of labeled bound ligand [28]. Experimental data were analyzed using Microsoft Excel (Microsoft Corporation). All plots had R^2^ values of at least 0.95.

### In vitro solubility assays

Series FLLL32 and FLLL62 neat standard solutions at the concentration of 0.1, 0.5, 1, 2, 5, 10, 20, 50, 100 µM in 50% acetonitrile with 0.1% formic acid were made from 20 mM FLLL32 and FLLL62 DMSO solutions. Triplicate 200 µl FLLL32 and FLLL62 solutions were made in cell culture media from DMSO solutions with the intended concentrations of 0.1, 0.5, 1, 2, 5, 10, 20, 50, 100 µM. In all solutions the final amount of DMSO was 0.25%. The FLLL32 and FLLL62 solutions in cell culture media were then centrifuged at 18,000× *g* at 4°C for 10 min and 100 µl of clear supernatant was removed to a clean centrifuge tube. FLLL32 and FLLL62 in neat standard solutions and cell culture supernatants were quantified using the validated LC/MS assay [30].

### Immunoblot Analysis

Lysates were prepared from cell lines using Laemmli buffer and assayed for protein expression by immunoblot analysis as previously described with antibodies (Ab) to STAT1 (BD Biosciences), pSTAT1, STAT3, pSTAT3, poly-ADP-ribose polymerase (PARP), Cyclin D1, Caspase-3, p-p38 MAPK (Thr180/Tyr182), p38 MAPK (clone 28B10), pAKT, AKT, phosphor-p44/42 MAPK (ERK1/2; Thr202/Tyr204) (Cell Signaling Technology, Danvers, MA), ERK2 (C-14 clone; Santa Cruz Biotechnology, Santa Cruz, CA) or β-actin (Sigma) [20]. Following incubation with the appropriate horseradish-peroxidase-conjugated secondary Ab, immune complexes were detected using the SuperSignal West Pico Chemiluminescent Substrate (Thermo Scientific/Pierce, Rockford IL).

### Annexin V/Propidium Iodide Staining

Phosphatidyl serine exposure was assessed in tumor cells by flow cytometry using APC-Annexin V and propidium iodide (PI; BD Pharmingen, San Diego, CA) as described [31]. Analyses were performed utilizing at least 10,000 events.

### Enzyme Linked Immunosorbent Assay (ELISA)

Human VEGF secretion was analyzed in RCC cell supernatants by commercially available Duo-Set ELISA according to manufacturer's recommendations (R&D Systems, Minneapolis MN).

### Real Time PCR

Real-time PCR was used to evaluate the expression of the IFN-γ stimulated gene (IRF1) as described [20], [32], [33] with pre-designed primer/probe sets (Assays On Demand; Applied Biosystems, Foster City, CA) and 2X TaqMan Universal PCR Master Mix (Applied Biosystems) per manufacturer's recommendations. Primer/probe sets for 18 s rRNA (Applied Biosystems) were used to normalize expression values (housekeeping gene). Data were acquired and analyzed using the ABI Prism 7900HT Sequence Detection System (Applied Biosystems).

### In vitro generation of MDSC

PBMC were isolated from normal donor source leukocytes via density gradient centrifugation with Ficoll-Paque, (Amersham Pharmacia Biotech) as described [34]. The protocol for *in vitro* generation of MDSC protocol was adapted from Lechner *et al.* [22], [23] as previously published by our group [24]. Briefly, cells were plated at a concentration of 5×10^5^ cells/mL in complete media (RPMI media supplemented with 10% fetal bovine serum and antibiotics). GM-CSF and IL-6, were added to the media at a concentration of 10 ng/mL in the presence of STAT3 inhibitors (FLLL32 or FLLL62) or vehicle control (DMSO). Cells were cultured at 37°C for 7 days, with media and cytokine/drug replacement every 2–3 days. After 7 days, suspension and adherent cells were harvested and myeloid cells were isolated from culture using the Easy Sep Myeloid Isolation Kit (Stem Cell Technologies). Cells were labeled with anti-CD33/66b magnetic microbeads and positively selected using an Easy Sep magnet. Isolated cells were washed twice prior to further studies. PBMC isolated from the same donor but not treated with cytokines were used as a control. Cells were analyzed using a BD FACScalibur flow cytometer using at least 10,000 cells in the lymphocyte gate based on light scatter properties. Appropriate intracellular and extracellular isotype control antibodies were used to control for background staining as described by our group [24].

### Statistical Analysis

The 4-parameter logistic or Hill model [35] was the assumed dose-response relationship between the compound concentration and the proportion of apoptotic cells. Nonlinear least squares regression (SAS version 9.2, SAS Institute) was used to estimate the parameters. Functions of the resulting parameter estimates were used to calculate and compare the absolute IC_50_ values for the compounds. Linear mixed effects models which included a random effect for experiment were used to analyze VEGF ELISA results. An interaction effect between compound and dose was included in the model to test for a differential effect of compound on VEGF secretion at the various doses.

## Results

### Small molecule Jak2-STAT3 pathway inhibitors FLLL32 and FLLL62

**Figure 1:**
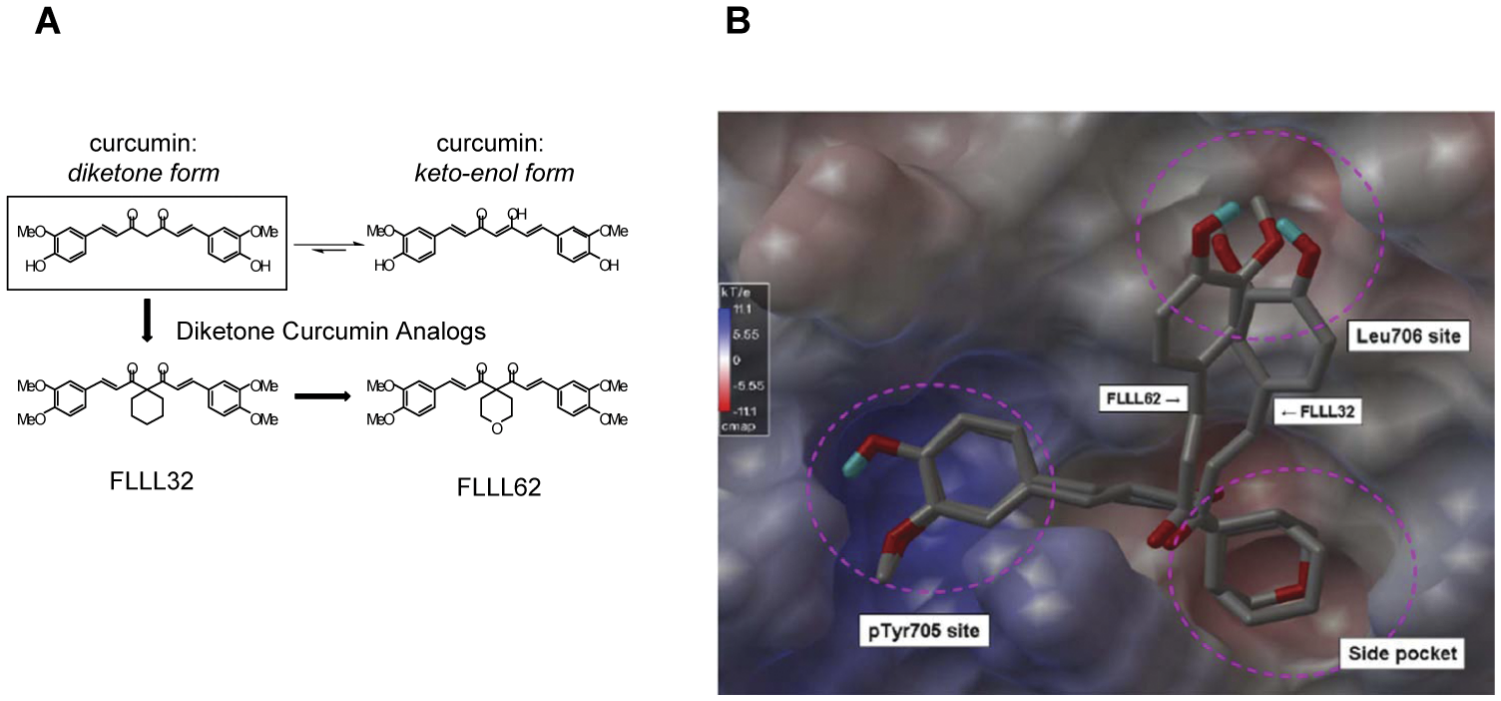
Molecular structure of FLLL32 and FLLL62 curcumin analogs. (A) Structural representation of curcumin and the FLLL32 and FLLL62 diketone analogs. (B) Computational representation of FLLL32 and FLLL62 binding to the STAT3 SH2 domain. The SH2 surface was shown with electrostatic potential, with blue representing positive charged surface and red representing negative charges. Both compounds bind to the pTyr705 site identically with a slight difference occurring at the Leu706 site.

FLLL32 and FLLL62 are small molecule inhibitors that were designed and modeled based on the diketone form of the natural product, curcumin. The two hydrogen atoms on the central carbon of curcumin were replaced with either a spiro-cyclohexyl ring (FLLL32) or an oxygen-containing spiro-tetrahydropyranyl ring (FLLL62) (Figure 1A). The presence of these moieties locks the molecule into the diketone from, and eliminates its ability to enolize. The oxygen-containing FLLL62 analog was considered an initial step for incorporation of additional hydrophilic groups into the FLLL32 scaffold to improve solubility, as addition of this atom lowers the calculated logP value of the molecule by approximately 1.5 (ChemDraw Ultra 11.0). These compounds have also been designed with replacement of the 3,4-hydroxyl groups with 3,4-dimethoxy groups. These modifications are predicted to block the sites on curcumin normally susceptible to glucuronidation, which in theory allows for greater metabolic stability. Structure-activity relationship studies using a docking approach indicate that FLLL32 and FLLL62 bind to the STAT3 SH2 domain almost identically (Figure 1B). Both compounds bind to a relatively hydrophobic side pocket besides the pTyr^705^ and the Leu^706^ binding subsites of STAT3 SH2. These interactions promote blocking homodimerization of Tyr^705^ phosphorylated STAT3 proteins and ensuing STAT3 activation. The slight difference in modeled binding modes occurs in binding to the subsite of Leu^706^. FLLL32 and FLLL62 also have similar direct binding potency to the STAT3 SH2 domain since the modeled binding free energies are −7.9 Kcal/mol and −8.0 Kcal/mol, respectively.

### Competitive efficiencies of FLLL32 and FLLL62 for inhibition of STAT3 dimerization in vitro

**Figure 2:**
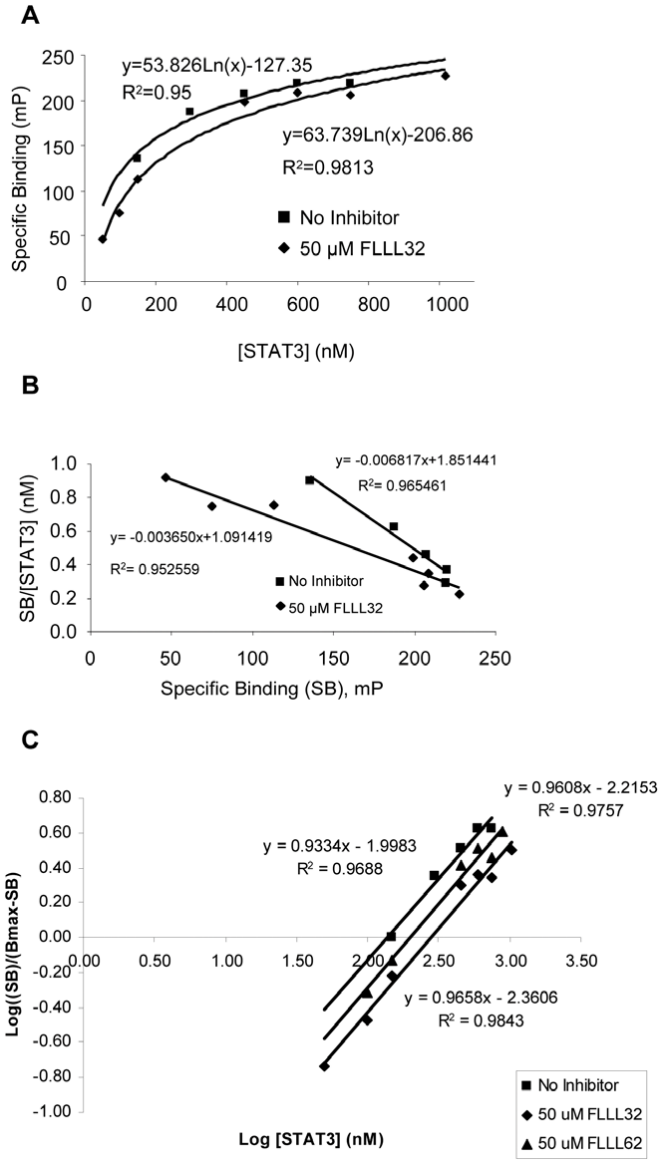
FLLL32 and FLLL62 inhibit STAT3 dimerization *in vitro*. (A) Saturation curves for fluorescence polarization assay. Data represent the change in specific binding observed when 4 nM 5-FAM-SpYLPQTV was incubated with increasing concentrations of STAT3 in the presence and absence of 50 µM FLLL32. (B) Scatchard Plot analysis of the binding of 4 nM 5-FAM-SpYLPQTV in the presence and absence of 50 µM FLLL32 over increasing concentrations of STAT3. Specific binding was plotted along the x-axis while the quotient of the specific binding divided by the corresponding concentration of STAT3 was plotted along the y-axis. The slope of each data set is equal to −1/K_d_ while the x-intercept is equal to B_max_. (C) Hill Plot analysis of the binding of 4 nM 5-FAM-SpYLPQTV in the presence and absence of 50 µM FLLL32 and FLLL62 over increasing concentrations of STAT3. The log of the quotient of the specific binding divided by the difference between the B_max_ and specific binding is plotted along the y-axis while the log of the corresponding concentration of STAT3 is plotted along the x-axis. A slope of 1 indicates noncooperativity in binding. A right-handed shift of the plot shows an increase in the K_d_.

A fluorescence polarization assay was validated to measure the direct effects of FLLL32 and FLLL62 on STAT3 dimerization *in vitro* (Figures S1, S2, S3). When no inhibitor was added, 4 nM 5-FAM-SpYLPQTV bound to STAT3 producing a K_d_ of 146.7 nM after a 24 hour incubation at room temperature. When 50 µM FLLL32 was present the saturation curve was shifted to the right (Figure 2A). It also resulted in a shift of the K_d_ to 274.0 nM; which was calculated via Scatchard Plot analysis (Figure 2B). The Hill Plot resulted in slopes of 0.9334 when no inhibitor was present and 0.9658 and 0.9608 in the presence of 50 µM FLLL32 and FLLL62, respectively (Figure 2C). These values are relatively close to 1, again suggesting that the binding is non-cooperative. The presence of 50 µM FLLL62 resulted in effects similar to those of FLLL32 (Figure 2C), but a smaller shift in the K_d_ value was observed (204.8 nM). This data suggests that both FLLL32 and FLLL62 have inhibitory activity, but that FLLL32 has a higher competitive efficiency. This data is consistent with what has been predicted by computational modeling. As expected neither FLLL32 nor FLLL62 showed inhibitory activity that was similar to that of SpYLPQTV, the high affinity peptide that binds the SH2 domain of STAT3.

### FLLL62 has an enhanced solubility profile as compared to FLLL32

**Figure 3:**
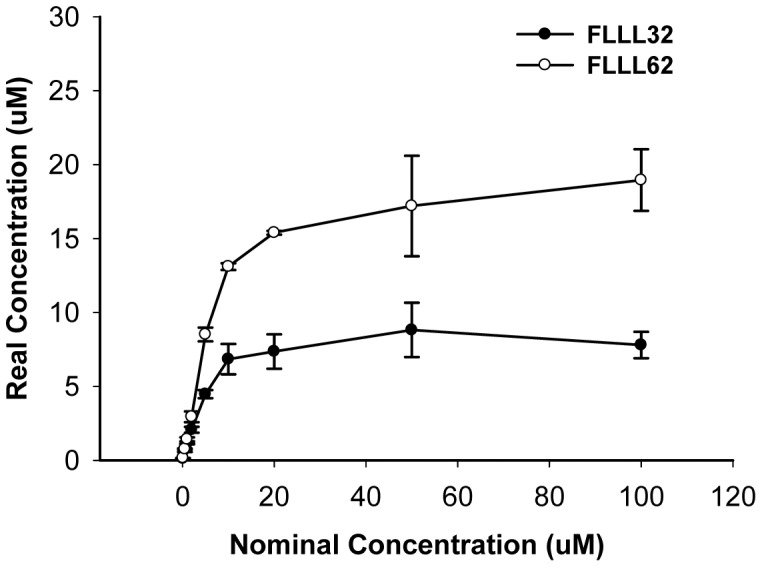
Solubility of FLLL32 and FLLL62 in cell culture media. Nominal concentration is the intended concentration of FLLL32 and FLLL62 solutions in cell culture media. Real concentration is FLLL 32 and FLLL 62 concentration in 100 µl supernatant which is calculated from FLLL32 and FLLL62 standard curves.

Standard curves of FLLL32 and FLLL62 were obtained from neat standard solutions and used for quantification of FLLL32 and FLLL62 in cell culture media supernatants. Results from these experiments indicated that both FLLL32 and FLLL62 could be completely dissolved in cell culture media at or below 5 and 10 µM, separately. As FLLL32 and FLLL62 concentrations were increased, the solubility was not increased proportionally and then saturated. However, the overall solubility of FLLL62 was approximately 2-fold higher as compared to that of FLLL32 (Figure 3).

### FLLL32 and FLLL62 induce apoptosis and inhibit STAT3 phosphorylation in RCC cell lines

**Figure 4:**
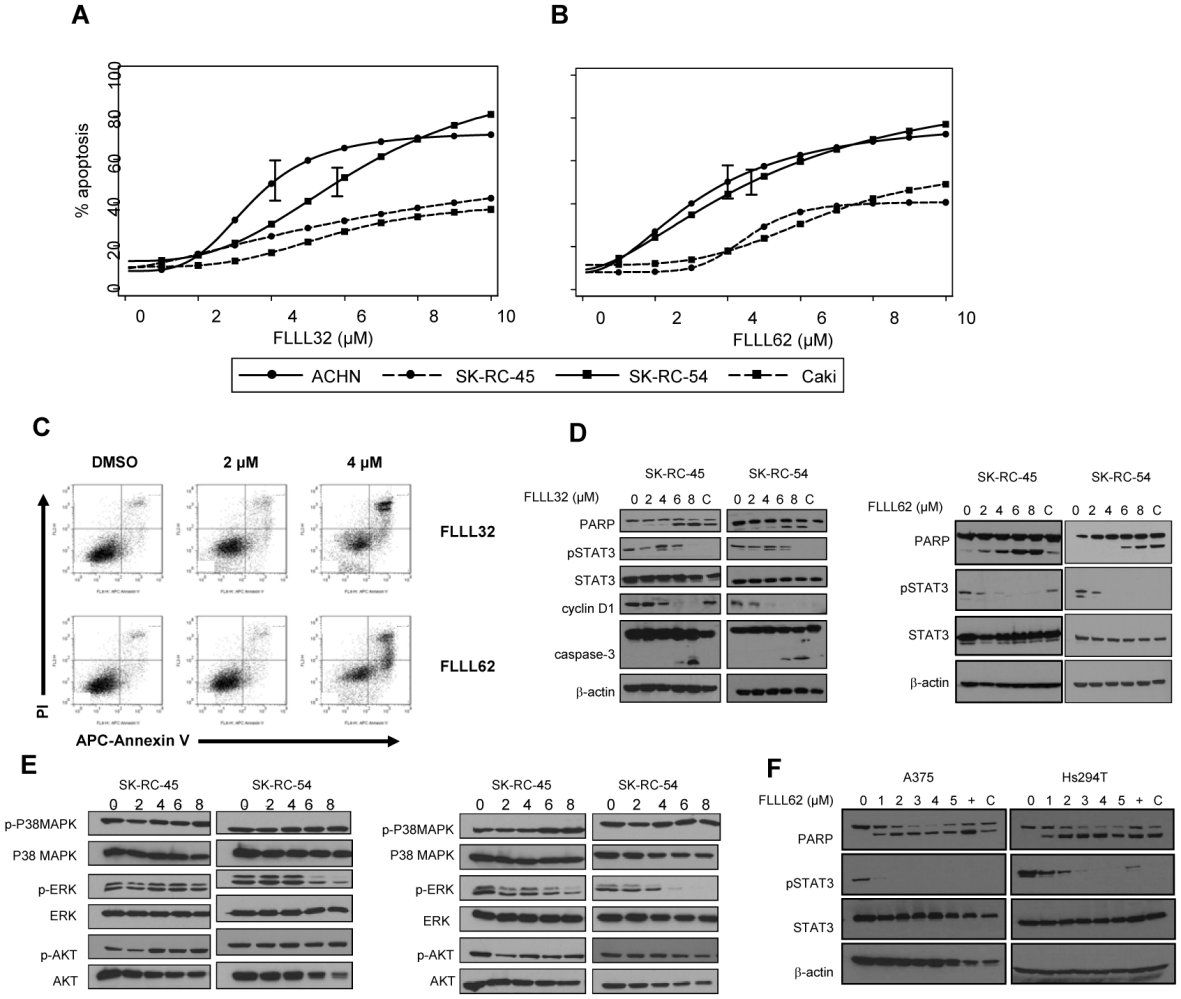
FLLL32 and FLLL62 induce apoptosis in human RCC cell lines. Annexin V/propidium iodide (Ann V/PI) staining of human RCC lines (Caki, ACHN, SK-RC-45, SK-RC-54) following a continuous 48 hour treatment with various doses of (A) FLLL32 or (B) FLLL62. Data were derived from at least three independent experiments. 95% confidence bounds are shown for the absolute IC_50_ estimates. (C) Representative flow cytometric analysis of apoptosis in human ACHN cells following a 48 hour treatment with DMSO (vehicle), FLLL32 or FLLL62. Inhibition of STAT3 phosphorylation (at Tyr^705^) was confirmed via immunoblot following a 24 hour treatment with (D) FLLL32 or FLLL62. Processing of PARP from its native to its cleaved form was also assessed as a marker of apoptosis. The STAT3 regulated gene, Cyclin D1 was also reduced by FLLL32. Membranes were probed for total STAT3 protein and β-actin to control for loading. (E) Immunoblot analysis of ERK, p38 MAPK and AKT phosphorylation in human RCC lines after a 48 hour treatment with FLLL32 or FLLL62. (F) Reduced STAT3-phosphorylation and PARP cleavage in human metastatic A375 and Hs294T melanoma cell lines following a 24 hour treatment with FLLL62.

The specificity and cytotoxic capacity of these curcumin analogs was evaluated in a panel of human RCC cell lines. The pro-apoptotic effects of FLLL32 and FLLL62 were quantified by flow cytometry following annexin V/PI staining and found to be concentration-dependent. Apoptosis was induced consistently but to variable degrees across the four individual human RCC lines for both compounds (Figure 4A–C). The absolute IC_50_ was not achieved in either SK-RC-45 or Caki, but ranged from 4.0 to 5.8 µM for ACHN and SK-RC-54 following a 48 hour treatment. In most cell lines tested, the FLLL32 and FLLL62 compounds induced a comparable degree of apoptosis. One exception was the SK-RC-54 cell line which had an absolute IC_50_ of 4.6 µM for FLLL62 and an absolute IC_50_ of 5.8 µM for FLLL32 (p = 0.015). Consistent with prior studies by our group, FLLL32 and FLLL62 were more potent than curcumin which required a 10-fold greater concentration to elicit the same pro-apoptotic effect *in vitro* [36]. The ability of FLLL32 and FLLL62 to inhibit STAT3 phosphorylation at Tyr^705^ was confirmed via immunoblot analysis in a panel of pSTAT3^+^ human RCC lines (Figure 4D and Figure S4). Treatment of the RCC cell lines also resulted in a reduced expression of the STAT3-regulated protein, cyclin D1 and increased processing of caspase-3 and PARP as compared to (DMSO) vehicle treated cells or curcumin (Figure 4D). The FLLL32 and FLLL62 compounds were relatively selective for inhibiting STAT3 Tyr^705^ phosphorylation as compared to phosphorylation of other representative pathways targeted by curcumin including ERK, AKT, and p38 MAPK (Figure 4E). There was some decrease in the level of pERK at higher doses (6–8 µM), particularly in the SK-RC-54 cell line after treatment with FLLL62. Although STAT3 phosphorylation was reduced at lower doses, we cannot completely exclude potential for some off-target effects, cross-talk between STAT3 and ERK, or even a caspase-mediated reduction in pERK due to the fact that cells were undergoing apoptosis. Importantly, FLLL32 and FLLL62 induced apoptosis in cell lines derived from other solid tumors including A375 and Hs294T metastatic melanoma (Figure 4F). These data confirmed that the activity of the inhibitors was not limited to only RCC cell lines, and could be employed against other malignant targets.

### IFN-γ-induced STAT1 phosphorylation is not adversely affected by FLLL32 or FLLL62

**Figure 5:**
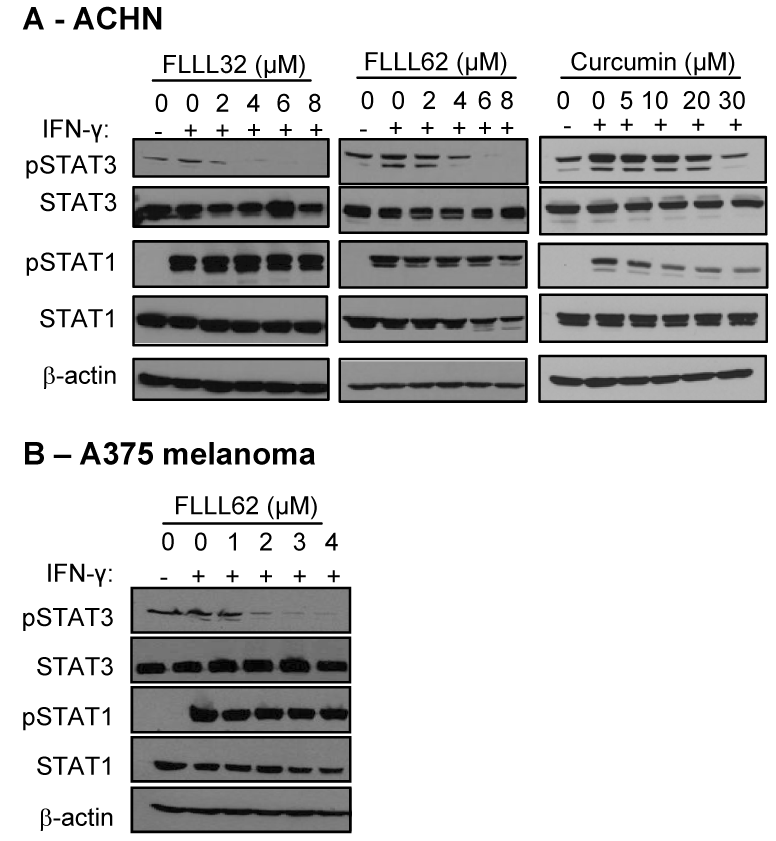
IFN-γ-induced signal transduction, in the presence of FLLL32 and FLLL62. (A) Human ACHN RCC cells or (B) A375 melanoma cells were pre-treated for 16 hours with DMSO (negative control), multiple doses of FLLL32, FLLL62, or curcumin and subsequently stimulated with IFN-γ (10 ng/mL) or PBS (vehicle) for 15 minutes. The expression of pSTAT1 and pSTAT3 were evaluated by immunoblot. Membranes were probed for total STAT3 protein, total STAT1 protein and β-actin to control for loading.

In contrast to STAT3, signal transduction mediated through the STAT1 transcription factor is thought to promote apoptosis and inhibit angiogenesis [37]–[39]. Thus, a truly specific STAT3 pathway inhibitor would not adversely affect the cellular response to interferon-gamma (IFN-γ), which mediates many of its cellular effects via phosphorylated STAT1 homodimers [32]. To test potential interactions between FLLL32 or FLLL62 and STAT1 in a biologic system, we investigated the effects of pre-treatment with these inhibitors on IFN-γ-induced signaling and gene expression of representative metastatic human RCC and melanoma cell lines. Pre-treatment of the pSTAT3 positive ACHN cell line with FLLL32 and FLLL62 STAT3 inhibitors for 16 hours, followed by a 15 minute stimulation with IFN-γ (10 ng/mL) led to reduced levels of pSTAT3 as compared to DMSO pre-treated cells (Figure 5A). However, pre-treatment with FLLL32 or FLLL62 did not adversely affect IFN-γ-induced pSTAT1 in human RCC cell lines (Figure 5A). A similar specificity for STAT3 was also observed in the A375 human melanoma cell line, as IFN-γ-induced pSTAT1 was not altered and basal pSTAT3 was reduced after pre-treatment with FLLL32 [20] or FLLL62 (Figure 5B). Similar to our prior studies in melanoma [36], curcumin pre-treatment required a much higher concentration to inhibit basal STAT3 phosphorylation and also reduced IFN-γ-induced pSTAT1 (Figure 5A).

### IFN-γ-induced gene expression is not adversely affected by FLLL32 but is reduced by FLLL62

**Figure 6:**
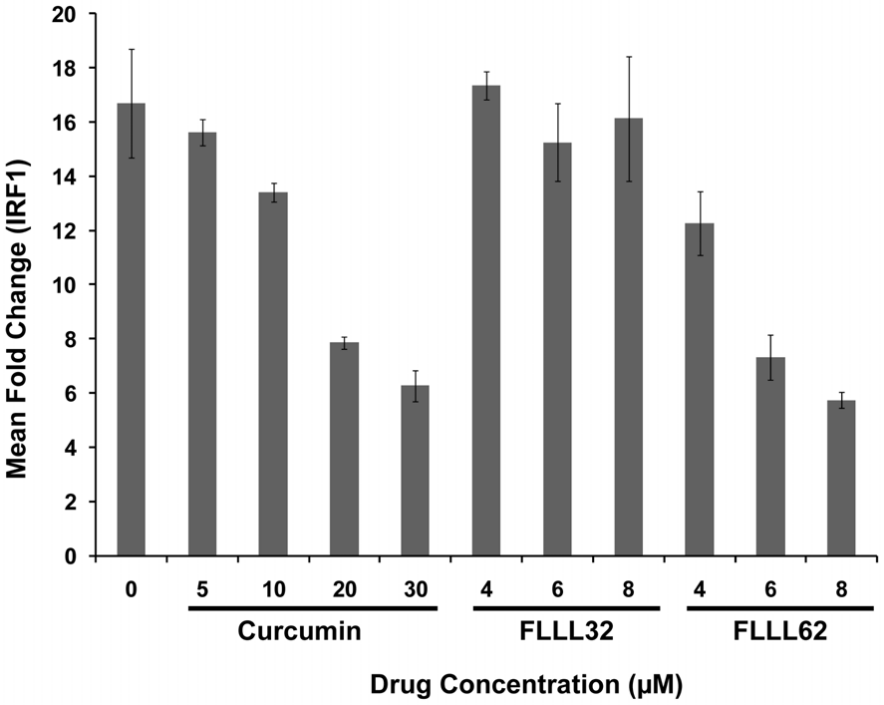
IFN-γ-induced gene expression in the presence of FLLL32 and FLLL62. The expression of the IFN-γ-stimulated gene, IRF1 was evaluated by Real Time PCR, following a 1 hour pre-treatment with either DMSO (vehicle), FLLL32, FLLL62 or curcumin at multiple concentrations and subsequent stimulation with IFN-γ (10 ng/mL) or PBS (vehicle) for an additional 4 hours in human ACHN RCC cell lines. The expression of the IFN-γ -stimulated gene, IRF1 was evaluated by Real Time PCR. All Real Time PCR data are expressed as fold change versus DMSO-pre-treated and PBS-stimulated cells and were normalized to 18 s rRNA levels (housekeeping gene). Error bars represent the standard deviation from at least two independent experiments.

We were next interested in determining whether the profile of IFN-γ induced signal transduction translated into downstream differences in cytokine-induced gene expression. Consistent with the signal transduction studies, pre-treatment of the ACHN RCC cell line with multiple concentrations of FLLL32 did not adversely affect transcription of the interferon-regulatory factor-1 (IRF1) gene in response to IFN-γ stimulation as determined by Real Time PCR (Figure 6). However, FLLL62 pre-treatment reduced the transcription of IRF1 expression following IFN-γ stimulation (Figure 6). This prototypical IFN-γ responsive gene has been shown to be transcribed via STAT1-STAT1 homodimers binding to a gamma-activated sequence (GAS) element [32]. Consistent with prior studies by our group, transcription of IRF1 in response to IFN-γ was reduced in cells pre-treated with curcumin at concentrations which are effective for reducing STAT3 phosphorylation (Figure 6 and Figure 5A). These data confirmed that structural modifications present on the FLLL32 compound did not adversely affect STAT3 specificity as compared to the parent compound, curcumin or the FLLL62 analog.

### FLLL32 and FLLL62 inhibit VEGF secretion in RCC cell lines

**Figure 7:**
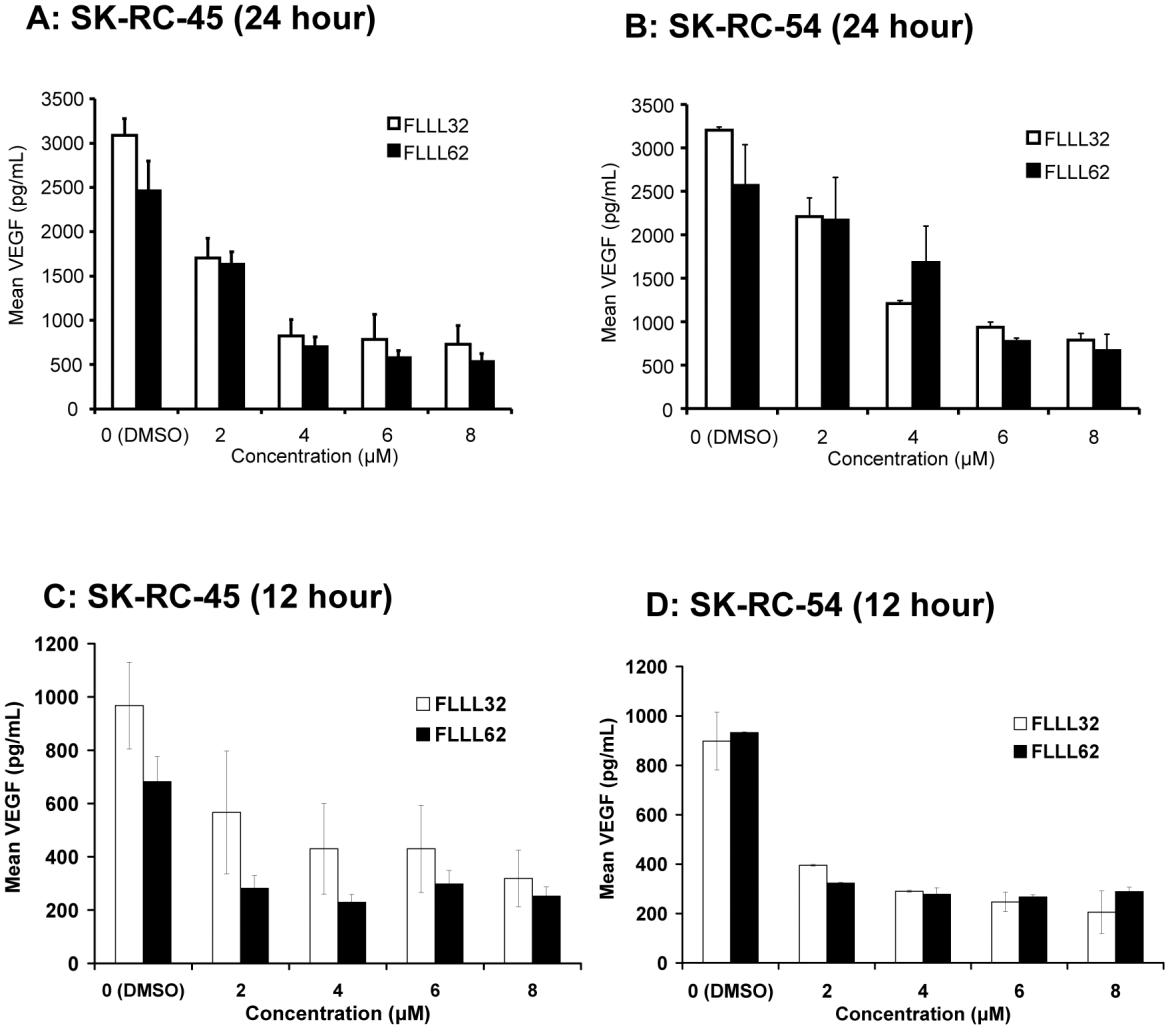
FLLL32 and FLLL62 inhibit VEGF Secretion in RCC cell lines. Soluble VEGF was measured in cell culture supernatants from SK-RC-45 and SK-RC-54 human RCC cultures via ELISA following a 24 hour (A–B) or 12 hour (C–D) treatment with FLLL32 or FLLL62. Error bars represent the standard deviation from three independent experiments.

STAT3 plays a key role in progression of renal cell carcinoma via inducing pro-angiogenic factors including VEGF [40], [41]. Soluble VEGF was measured in culture supernatants from human SK-RC-45 and SK-RC-54 RCC lines following a 24 hour treatment with FLLL32 or FLLL62 at concentrations ranging from 2–8 µM. These data revealed a significant decrease in VEGF secretion by FLLL32 (p<0.0001 for doses 2 µM–8 µM) and FLLL62 (p<0.005 for doses 4 µM–8 µM) as compared to vehicle (DMSO) treated cells (Figure 7A–B). The ability of FLLL32 vs. FLLL62 to inhibit VEGF secretion was not significantly different in these two cell lines at the same dose (p>0.05). The effect of FLLL32 and FLLL62 could not be determined in the ACHN and Caki cell lines as the level of VEGF secretion from these cells was very low at basal levels (not shown). The reduction in VEGF was also evident after treatment with the inhibitors for a shorter duration of 12 hours (Figure 7C–D). These data indicate that the reduced VEGF secretion is likely occurring on a cellular basis rather than due to profound apoptosis of bulk cell cultures.

### FLLL32 and FLLL62 inhibit in vitro generation of myeloid-derived suppressor cells

**Figure 8:**
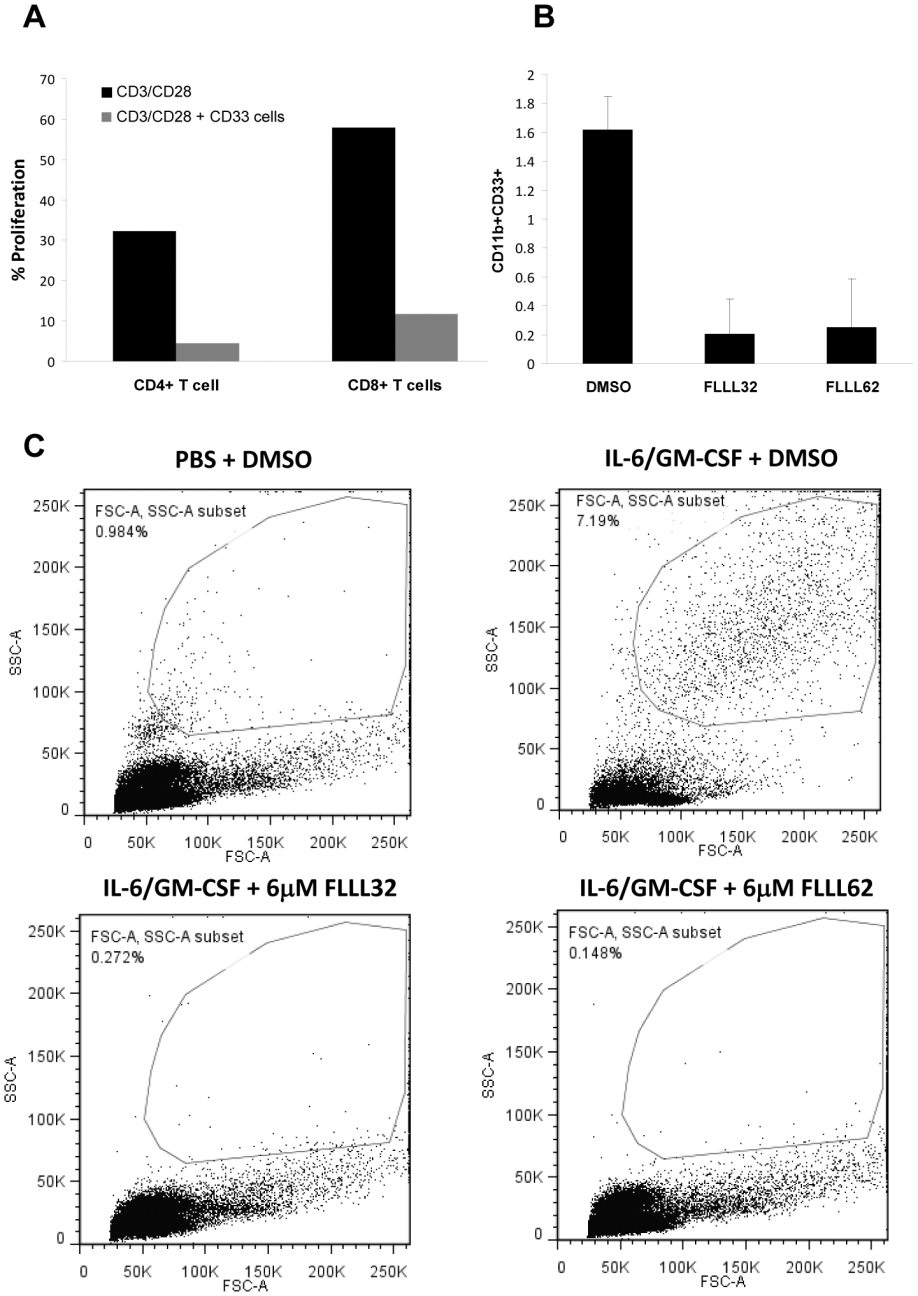
FLLL32 and FLLL62 inhibit IL-6 and GM-CSF mediated MDSC expansion. (A) MDSC were generated *in vitro* using PBMC from healthy adult donors cultured with IL-6 and GM-CSF (10 ng/mL each) for 7 days. MDSC were defined via a CD33^+^CD11b^+^ phenotype by flow cytometry as previously described [22]–[24]. (A) *In vitro* generated, CD33^+^ MDSC were isolated and confirmed functional through their ability to suppress proliferation of activated, autologous CFSE-labeled CD4^+^ and CD8^+^ T cells. Data shown are from a representative donor. (B) The presence of 6 µM FLLL32 or FLLL62 in the 7 day cultures inhibits generation of CD33^+^CD11b^+^ MDSC. Data are presented as the mean percentage of CD33^+^CD11b^+^ cells from culture. Error bars represent the standard deviation from two separate donors. (C) Forward and side scatter profiles from a representative donor are shown to illustrate the reduction in large granular cells.

In addition to its multiple roles within the tumor cell, STAT3 can promote immunosuppression in a tumor-bearing host via its ability to induce the differentiation of early myeloid cells into that of a myeloid-derived suppressor cell (MDSC) phenotype [42]. Therefore, the effects of FLLL32 and FLLL62 on the generation of MDSC from healthy donor PBMCs cultured with IL-6 and GM-CSF were evaluated *in vitro* as described previously [22]–[24]. We initially confirmed the MDSC generated using this method were functionally suppressive against autologous T cells stimulated to proliferate using ligation of CD3 and CD28 (Figure 8A). Addition of FLLL32 or FLLL62 to the 7-day differentiation cultures at 6 µM inhibited the expansion of MDSC with a CD33^+^CD11b^+^ phenotype (Figure 8B). This inhibitory effect was dramatic, as even examination of forward and side light scatter properties of cells revealed a clear reduction of large granular cells induced by IL-6 and GM-CSF (Figure 8C).

## Discussion

In the present study we demonstrate that the structure of a novel curcumin analog, FLLL32 can be modified to improve its solubility profile without compromising much of its functional activity or specificity. Both the FLLL32 lead compound and the FLLL62 compound directly interfered with STAT3 homodimerization in fluorescence polarization assays. Thus, our *in vitro* studies indicated the compounds may act as direct inhibitors of the STAT3 SH2 domain. For the first time, we also demonstrate that these curcumin analogs can induce apoptosis via STAT3 inhibition in human RCC cell lines and inhibit the release of soluble VEGF. Both FLLL32 and FLLL62 displayed specificity for STAT3 as compared to the homologous STAT1 protein and were relatively selective for STAT3 over other pathways targeted by the parent compound, curcumin. Finally, inhibition of STAT3 with these compounds led to reduced *in vitro* expansion of MDSC from PBMCs cultured in the presence of IL-6 and GM-CSF. These studies indicate that STAT3 pathway inhibition induces anti-tumor effects against human RCC and melanoma cell lines and has potential to reduce MDSC expansion.

Structural modifications to generate the FLLL62 compound resulted in an improved solubility profile. However, this appeared to be at the cost of some specificity as indicated by Real Time PCR analysis of IRF1, an IFN-responsive gene. Although the incorporation of the oxygen within FLLL62 did not alter initial IFN-γ-mediated STAT1 phosphorylation, our data suggest that it may disrupt the kinetics or duration of molecular interactions required for optimal IRF1 expression. These data illustrate how subtle changes in molecular structure can alter drug specificity. Whether or not the improvement in solubility is worthwhile despite the reduced specificity for STAT3 over STAT1 will need to be investigated in future *in vivo* studies.

The development of potent and specific inhibitors targeting the STAT3 protein directly is met with a number of challenges. First, there is a high degree of homology present between individual STAT proteins. This makes accurate targeting of STAT3 specific residues quite complicated. Second, the hydrophobic interactions responsible for STAT3 homodimerization have proven difficult to antagonize. Our group has taken advantage of the hydrophobic properties of the natural product, curcumin, and used it as a lead compound to develop small molecules capable of disrupting the STAT3 SH2 domain and subsequent dimerization [20], [21]. The parent compound, curcumin has been shown to inhibit phosphorylation of STAT3 in a variety of human cell lines including melanoma, pancreatic, and biliary cancer among others, albeit at higher concentrations [43], [44]. Importantly, curcumin can alter phosphorylation events on a number of cellular proteins involved in various cellular processes [45]. In the present study, a molecular docking approach allowed us to model the binding of both tautomeric forms of curcumin to the STAT3 SH2 domain, and identify relevant changes predicted to enhance STAT3 specificity. The current data suggests that the molecular scaffold of curcumin can be re-focused toward the most relevant pro-oncogenic targets.

The STAT3 pathway is an emerging oncogenic target in the setting of RCC, melanoma, and other forms of cancer (Reviewed in [46]). This molecular pathway is attractive for design of small molecule inhibitors due to the fact that it represents a point at which multiple extracellular signals converge to promote cell proliferation, metastasis, angiogenesis and immune suppression [19], [46]. Indeed, recent studies show that human RCC cell lines are sensitive to Jak2-STAT3 pathway inhibition by WP1066 [13] or dimethoxycurcumin [47]. Recent clinical data further suggest that agents regularly used in the therapy of RCC may elicit their anti-tumor effect via targeting the STAT3 pathway. For example, the broad acting kinase inhibitor sunitinib has recently been shown to elicit anti-tumor activity by STAT3 inhibition [48]. These findings suggest that targeted inhibition of the STAT3 pathway may be beneficial for patients with RCC or other types of cancer.

Although FLLL32 and FLLL62 induced cell death across all human RCC examined, their pro-apoptotic effects appeared somewhat variable depending upon the cell line. In fact, in the Caki and SK-RC-45 cell lines, we were not able to accurately determine the absolute IC_50_, even in the presence of 30 µM of drug for 48 hours. These findings suggest that the STAT3 pathway may function differently to regulate cellular survival within individual RCC cell lines. Despite these results, STAT3 inhibition remained an effective means to reduce VEGF secretion and growth inhibition in the SK-RC-45 cell line (Figure 7). These data indicate that STAT3 inhibitors could elicit a variety of beneficial anti-tumor effects. Certainly the biologic characteristics of each tumor type likely influence their response to STAT3 inhibition.

Recent data suggest that STAT3 inhibitors may be a useful means by which to compliment immunotherapeutic agents. In addition to its ability to influence the biology of malignant cells, STAT3 plays an important role in regulating the expansion and survival of immunoregulatory cell populations such as myeloid derived suppressor cells, T regulatory cells and Th17 cells [49]–[52]. These cell populations are markedly upregulated in patients with RCC or other tumors, and act to inhibit T and NK cell cytotoxic responses against malignant cells [50], [53], [54]. Indeed, our data demonstrate for the first time that small molecule STAT3 inhibitors can limit the ability of GM-CSF and IL-6 to induce expansion of MDSC *in vitro*. Multiple publications support using STAT3 inhibition in combination with immunotherapeutic regimens such as interferon-alpha, adoptive T cell transfer, or CpG oligodeoxynucleotides [16]–[19]. Importantly, prior studies by our group have shown that the FLLL32 analog does not induce apoptosis of PBMCs or NK cells cultured in the presence of IL-2 despite its potent direct effects on melanoma cells [20]. In further support of STAT3 inhibition as a means to augment immunotherapy was a study by Ito *et al* performed in patients with RCC. In this report, linkage disequilibrium mapping revealed that a SNP in the 5′ region of STAT3, rs4796793, leads to reduced STAT3 protein expression. Importantly this SNP was a significant predictor of clinical response to IFN-α. The authors of this study concluded that patients with a minor rs4796793 allele had better intrinsic immunosurveillance [55]. These data support the multiple cellular and systemic targets that may be modulated by STAT3 inhibition and the opportunity for enhancement of immunologic therapy.

In conclusion, these data demonstrate that potent and specific lead compounds can be generated from natural products, such as curcumin for inhibition of the STAT3 pathway. Because of their ability to directly interact with the STAT3 SH2 domain, elicit cytotoxic activity on human RCC cells, and inhibit MDSC expansion, this class of compounds will serve as viable lead compounds for future drug development.

## Supporting Information

Figure S1 Saturation curves for Change in Millipolarization (mP) at various concentrations of STAT3 with a constant concentration of fluorescent peptide (4 nM). **Validation of the Fluorescent Polarization Assay to measure STAT3 dimerization.** The saturation curves in the presence and absence of 8 nM SpYLPQTV (4 nM 5-FAM-SpYLPQTV was held constant) resulted in a pronounced shift to the right when 8 nM of the unlabeled peptide was present (Figure S1). The K_d_ values were calculated by Scatchard Plot analysis and determined to be 163.7 nM in the absence of SpYLPQTV and 823.0 nM when SpYLPQTV was present to compete with the STAT3 SH2 domain binding site (Figure S2). This large shift in the K_d_ demonstrates that the unlabeled peptide inhibits the binding of the fluorescent probe to the STAT3 SH2 domain and that the assay is functioning properly. Such a pronounced shift should be expected since the unlabeled phosphorylated peptide is expected to have a binding affinity to STAT3 that is similar to that of the fluorescent peptide. In the absence and presence of 8 nM SpYLPQTV the calculated B_max_ values from the Scatchard Plots were 191.0 and 194.8 mP, respectively, which demonstrates consistency. The slopes of the constructed Hill Plots were 0.9985 and 1.001, respectively, demonstrating that the binding is non-cooperative (Figure S3).
(DOCX)

Figure S2 Scatchard Plot used to calculate Kd values in the presence or absence of SpYLPQTV. **Validation of the Fluorescent Polarization Assay to measure STAT3 dimerization.** The saturation curves in the presence and absence of 8 nM SpYLPQTV (4 nM 5-FAM-SpYLPQTV was held constant) resulted in a pronounced shift to the right when 8 nM of the unlabeled peptide was present (Figure S1). The K_d_ values were calculated by Scatchard Plot analysis and determined to be 163.7 nM in the absence of SpYLPQTV and 823.0 nM when SpYLPQTV was present to compete with the STAT3 SH2 domain binding site (Figure S2). This large shift in the K_d_ demonstrates that the unlabeled peptide inhibits the binding of the fluorescent probe to the STAT3 SH2 domain and that the assay is functioning properly. Such a pronounced shift should be expected since the unlabeled phosphorylated peptide is expected to have a binding affinity to STAT3 that is similar to that of the fluorescent peptide. In the absence and presence of 8 nM SpYLPQTV the calculated B_max_ values from the Scatchard Plots were 191.0 and 194.8 mP, respectively, which demonstrates consistency. The slopes of the constructed Hill Plots were 0.9985 and 1.001, respectively, demonstrating that the binding is non-cooperative (Figure S3).
(DOCX)

Figure S3 Hill plots to assess binding. **Validation of the Fluorescent Polarization Assay to measure STAT3 dimerization.** The saturation curves in the presence and absence of 8 nM SpYLPQTV (4 nM 5-FAM-SpYLPQTV was held constant) resulted in a pronounced shift to the right when 8 nM of the unlabeled peptide was present (Figure S1). The K_d_ values were calculated by Scatchard Plot analysis and determined to be 163.7 nM in the absence of SpYLPQTV and 823.0 nM when SpYLPQTV was present to compete with the STAT3 SH2 domain binding site (Figure S2). This large shift in the K_d_ demonstrates that the unlabeled peptide inhibits the binding of the fluorescent probe to the STAT3 SH2 domain and that the assay is functioning properly. Such a pronounced shift should be expected since the unlabeled phosphorylated peptide is expected to have a binding affinity to STAT3 that is similar to that of the fluorescent peptide. In the absence and presence of 8 nM SpYLPQTV the calculated B_max_ values from the Scatchard Plots were 191.0 and 194.8 mP, respectively, which demonstrates consistency. The slopes of the constructed Hill Plots were 0.9985 and 1.001, respectively, demonstrating that the binding is non-cooperative (Figure S3).
(DOCX)

Figure S4 Immunoblot analysis of FLLL32 and FLLL62 on ACHN and Caki cell lines. **Effects of FLLL32 and FLLL62 on additional human RCC cell lines.** Inhibition of STAT3 phosphorylation (at Tyr^705^) was confirmed via immunoblot following a 24 hour treatment of ACHN and Caki human RCC cell lines with FLLL32 or FLLL62 (Figure SL4). Processing of PARP from its native to its cleaved form was also assessed as a marker of apoptosis. The STAT3 regulated gene, Cyclin D1 was also reduced by FLLL32. Membranes were probed for total STAT3 protein and β-actin to control for loading.
(DOCX)
