# Supplementary material for: Structurally Modified Curcumin Analogs Inhibit STAT3 Phosphorylation and Promote Apoptosis of Human Renal Cell Carcinoma and Melanoma Cell Lines
Source: PLoS One. 2012 Aug 10;7(8):e40724. doi: 10.1371/journal.pone.0040724 (PMC3416819; doi:10.1371/journal.pone.0040724)
Supplement: Supplementary file 2 [file pone.0040724.s002.docx]

S2
