# Supplementary material for: Structurally Modified Curcumin Analogs Inhibit STAT3 Phosphorylation and Promote Apoptosis of Human Renal Cell Carcinoma and Melanoma Cell Lines
Source: PLoS One. 2012 Aug 10;7(8):e40724. doi: 10.1371/journal.pone.0040724 (PMC3416819; doi:10.1371/journal.pone.0040724)
Supplement: Supplementary file 4 [file pone.0040724.s004.docx]

S4

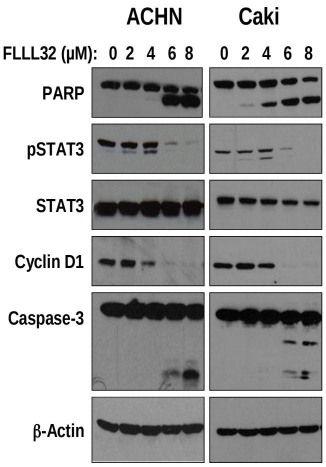
ACHN
Caki
FLLL32 (μM): 0 2 4 6 8 0 2 4 6 8
PARP
pSTAT3
STAT3
Cyclin D1
Caspase-3
β-Actin

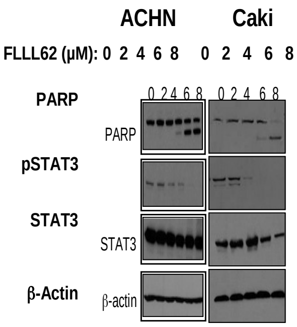
ACHN
Caki
FLLL62 (μM): 0 2 4 6 8 0 2 4 6 8
PARP
pSTAT3
STAT3
β-Actin
0 2 4 6 8
0 2 4 6 8
PARP
STAT3
β-actin
